# Evaluation of the synergistic efficiency of hormonal, biological and physical stimuli in the induction and growth of callus in lavender

**DOI:** 10.12688/f1000research.173637.1

**Published:** 2026-03-18

**Authors:** Mohammed Abdulgafor Mohammed, Masara Dafer Alani, Ahmed O. Mashaan, Ali F. Almehemdi

**Affiliations:** 1 Department of Biotechnology, University of Anbar, Ramadi, 31001, Iraq; 2 Department of Studies and Planning, University of Anbar, Ramadi, 31001, Iraq; 3 Center of Desert Studies, Department of conservation Agriculture, University of Anbar, Ramadi, 31001, Iraq

**Keywords:** Lavender, induction callus, biostimulants, hormonal, physical, biological.

## Abstract

**Background:**

Lavender is a crucial source of secondary metabolites with medicinal and industrial importance. Its exploitation faces challenges due to the limited availability of raw materials and their sensitivity to climate. Tissue culture techniques, particularly callus culture, represent the most sustainable method for the continuous production of these active compounds.

**Methods:**

This study focused on developing an effective protocol for inducing and stimulating callus cultures in lavender plants. In the growth phase, different concentrations of 2,4-D and benzyladenine were assessed. In the induction phase, different concentrations of the growth regulator 2, 4-D (0, 1, 2 and 3) mg L ^−1^ were tested under light and dark conditions. The experimental approach investigated three primary variables hormone concentration, environmental conditions and the type of nutrient medium. A comparative analysis was also completed between two nutrient media, MS and B5, supplemented with different Chitosan concentrations (0, 50, 100 and 200) mg. L ^−1^.

**Results:**

The results indicated that the optimal concentration for callus induction was 1 mg L ^−1^ of 2,4-D, with the highest callus formation rate 95% recorded under dark conditions. Among all treatments, the combination of 2, 4-D 1 mg L ^−1^ and BA 2 mg L ^−1^ was the most effective for sustainable callus growth. Regarding the nutrient medium, B5 was more efficient than the MS medium. For the biostimulants, Chitosan at 100 mg L ^−1^ recorded the highest efficiency in both fresh and dry callus weights.

**Conclusions:**

This study underscores the necessity of integrating hormonal, nutritional and environmental factors for optimal callus production.

## Introduction

Lavender ( *Lavandula angustifolia* Mill.) is a member of the Lamiaceae family of flowering plants and is the most widely cultivated of the family, which also has over 39 other genera ( Vârban *et al*., 2022, Kumar *et al*., 2025). The varied secondary metabolites in lavender provide a broad range of industrial uses in the pharmaceutical, cosmetic, and food industries ( Crisan *et al*., 2023). The medicinal value of lavender has been well established over the centuries, considering the wide range of advanced herbal diseases and its other numerous pharmacological attributes, including antimicrobial, antifungal, antispasmodic and other active antioxidant ( Hassiotis and Vlachonasios, 2025) as well as digestive function regulating ( Firoozeei *et al.*, 2021) properties. It is also used to treat gastrointestinal disorders, cardiovascular diseases, and respiratory and urinary disorders, anxiety, depression, and sleep disorders ( Kumar *et al*., 2025), mood swings ( Mehrabian *et al*., 2022), chronic pain ( Pehlivan and Karadakovan, 2019), restless legs syndrome ( Ghasemi *et al*., 2021), migraines ( Yuan *et al*., 2021), and behavioural disorders in dementia patients. ( Scuteri et al., 2019) as well as improving physical symptoms such as blood pressure and respiratory rate ( Contrada *et al*., 2021).

There are difficulties in the plant based pharmaceutical industry related to the sourcing of raw materials attributable to the isolation of active compounds being arduous due to plants being low in active compounds or active compounds being affected by climatic conditions. Under these circumstances, the cultivation of plant tissues offers exciting possibilities, as it promotes the incessant active compounds synthesis irrespective of the season. This facilitates the overcoming of climatic and geographical limitations, hence aiding in the supply of growing the demand for medicinal and aromatic plants. This is one of the refined and improved strategies in the biotechnological domain that aims or is directed towards the amplified generation of secondary metabolites relevant for the industry ( Ozyigit *et al*., 2023). This necessitates the adjustment and calibration of every single component in every single process of culture tissue namely, nutrient medium, the different and varied growth hormones, and the different types of stimulants to maximise biomass and secondary metabolites ( Adil *et al*., 2025). The technology of callus culture is one of the sustainable methods for the valuable plant compounds and for the preservation of the habitat of the medicinal plants ( Abed *et al*., 2020, Babich *et al*., 2020, Alrawi *et al*., 2022). Compounds can be derived from callus tissue in a lab setting without having to use whole plants, and this method can be commercially scaled by employing bioreactors for compound bioprocessing. in vitro callus tissue development depends on a number of factors; for example, a plant’s genetics, the plant organ type, and the composition of the nutrient medium and the growth regulators ( Miri and Roughani, 2018). Furthermore, callus culture technology depends on the growth and sustained production and the qualitative and quantitative production of active compounds. The optimum environment must also be available. The importance of growth regulators during callus formation is significant because, in some cases, a tissue may be unresponsive or may even die if the medium’s hormone levels are set higher or lower than the tissue’s internal level ( Yegorova *et al*., 2020). Previous research indicates that the callus formation is more pronounced when the nutrient medium is enhanced with growth regulators, specifically auxins and cytokinins ( Jabr and Mohsen, 2024). The optimization of the hormonal milieu is only attained with the moderators acting synergistically within set ranges. Compared to using each of them separately ( Tilkat and Onay, 2009). The balance between auxins and cytokinins in the nutrient medium is a key determinant that stimulates cell division and initiates callus formation. In addition, biostimulants such as chitosan can enhance biomass production and secondary metabolites in plant tissue cultures ( Javed *et al*., 2025). It acts as a plant defence enhancer capable of activating various physiological and biochemical processes in different plant species ( Iber *et al*., 2022). Chitosan is a biopolymer derived from chitin, which can be produced economically by recycling the exoskeletons of crustaceans. Given the notable lack of studies on callus induction in lavender, this study aims to evaluate the efficiency of biological and physical stimuli in inducing callus and promoting its growth in this plant.

## Materials and methods

### Plant materials

Lavender seeds were obtained from a specialised seed production company Al-Wadi seed Company located south of the capital Baghdad, Iraq. The seeds were stored in a laboratory at a temperature of 15°±2 C until use.

### Establishment of tissue culture

A nutrient medium consisting of half MS salts ( Murashige and Skoog, 1962) and gibberellic acid at a concentration of 0.5 mg L ^−1^ and 30 g L ^−1^ sucrose was prepared. The solution was then transferred to a graduated cylinder and the volume was made up to 1000 mL with sterile distilled water. The pH of the medium was adjusted to 5.7 ± 0.1 using NaOH or HCl solutions at a concentration of (0.1). 7 g. per litre of agar was added as a solidifying agent. The medium was mixed thoroughly and heated with stirring until it reached full boiling point (100° C) to ensure that the agar dissolved.

The medium was distributed into glass culture vessels 10 ml vessel. The containers were sealed with tight fitting lids and sterilised in an autoclave Model ABC at a temperature of 121° C and a pressure of 1.04 kg cm for 15 minutes. The sterilised medium was stored at 25° C until use. The seeds were sterilised according to the method used by the researcher, whereby the seeds were washed with running water to remove floating seeds and dust. The healthy seeds were transferred to the laminar air cabinet, then a 3% sodium hypochlorite sterilising agent was added to the seeds for 15 minutes with continuous stirring ( Al-Alwani and Mohammed, 2022).

The seeds were rinsed with distilled water for 5 minutes and the process was repeated three times to ensure that the sterilising agent was removed. The sterilised seeds were planted on the prepared nutrient medium. The planted seeds were then transferred to the growth chamber at a temperature of 25° C and a light intensity of 1000 lux for 16 hours of light and 8 hours of darkness ( Sekhi et al., 2025). After four weeks, seedlings ready for use were obtained for callus induction experiments. The chemicals listed in Table 1 were used in this study.

**Table 1.** Reagents used in the study, including the amount used, supplier information, and catalogue numbers.

Reagent item	Quantity	Supplier	Catalogue numbers
MS.	4.74 g. L ^−1^	Duchefa Biochemie	M0221
Gamborg B5 medium	3.92 g. L ^−1^	Duchefa Biochemie (Holland)	G0209
Dichlorophenoxyacetic acid	(0,1,2 and 3) mg. L ^−1^	Duchefa Biochemie (Holland)	D0911
Benzyladenine	BA (0,1 and 2) mg. L ^−1^	CDH (India)	022625
Sucrose	30 g. L ^−1^	CDH (India)	030299
Chitosan	(0,50,100, 200) mg. L ^−1^	CDH (India)	046555
Agar Agar	7 g. L ^−1^	Duchefa Biochemie (Holland)	P1001.1000

### Callus induction

A nutrient medium based on full MS salts strength was prepared, with varying concentrations of the growth regulator dichlorophenoxyacetic acid (2,4-D) (0, 1, 2 and 3) mg L ^−1^. Then 30 g L ^−1^ of sucrose was added to the medium as a carbon source and 7 g L ^−1^ of agar as a stabilising agent. The nutrient medium was then sterilised according to the standard protocol. After preparing the media, parts of the leaves were excised and then planted on the prepared nutrient media containing the specified concentrations of the growth regulator 2,4-D. The cultures were incubated in a growth chamber with controlled conditions at a constant temperature of 25 C. To investigate the effect of light on callus induction, the cultures were exposed to two different lighting regimes complete darkness and 1000 lux light intensity under a cyclic light regime 16 hours of light 8 hours of darkness. At the end of the four-week incubation period, data were collected by measuring Induction rate the percentage of tissue sections in which callus was formed, Fresh weight and Dry weight.

The nutrient medium was prepared mainly from full-MS salts strength at a concentration of 4.74 g L ^−1^, with the addition of 30 g L ^−1^ sucrose and 7 g L ^−1^ agar. This medium was then enriched with varying concentrations of auxin 2,4-D at levels of (1, 2, and 3) mg L ^−1^ combined with concentrations of cytokinin benzyladenine BA at levels of (0, 1 and 2) mg L ^−1^. After sterilising the media, callus pieces of a fixed weight 150 mg were transferred to it three days after preparation. The cultures were incubated in complete darkness at a constant temperature of 25° C for four weeks. At the end of the incubation period, data were collected by measuring the fresh weight and then the dry weight of the callus growth.

### The role of nutrient components and chitosan in promoting callus growth

Two types of nutrient media MS and B5 were used, prepared with different concentrations of chitosan (0, 50, 100 and 200) mg L ^−1^, with the addition of 30 g L ^−1^ sucrose and 7 g L ^−1^ agar. Callus pieces weighing 150 mg were transferred to these sterile media containing different concentrations of chitosan. After a four-week incubation period, growth rates were determined by measuring the fresh and dry weights of the callus formed.

### Statistical analysis

The data were statistically analysed using GenStat statistical software (Gen 12 ed) ( Payne *et al*., 2009) according to a completely randomised design (C.R.D.), with ten replicates for each treatment. The treatments were arranged in a factorial arrangement. The means were compared on LSD test after partitioning the variance components via fisher's test by using probability ≤ 0.05 whereas, the callus pecentage was analysed using originPro 2025b ( origin 2025).

## Results and discussion

### The effect of light conditions and 2,4-D concentrations on callus formation

**Figure 1.**
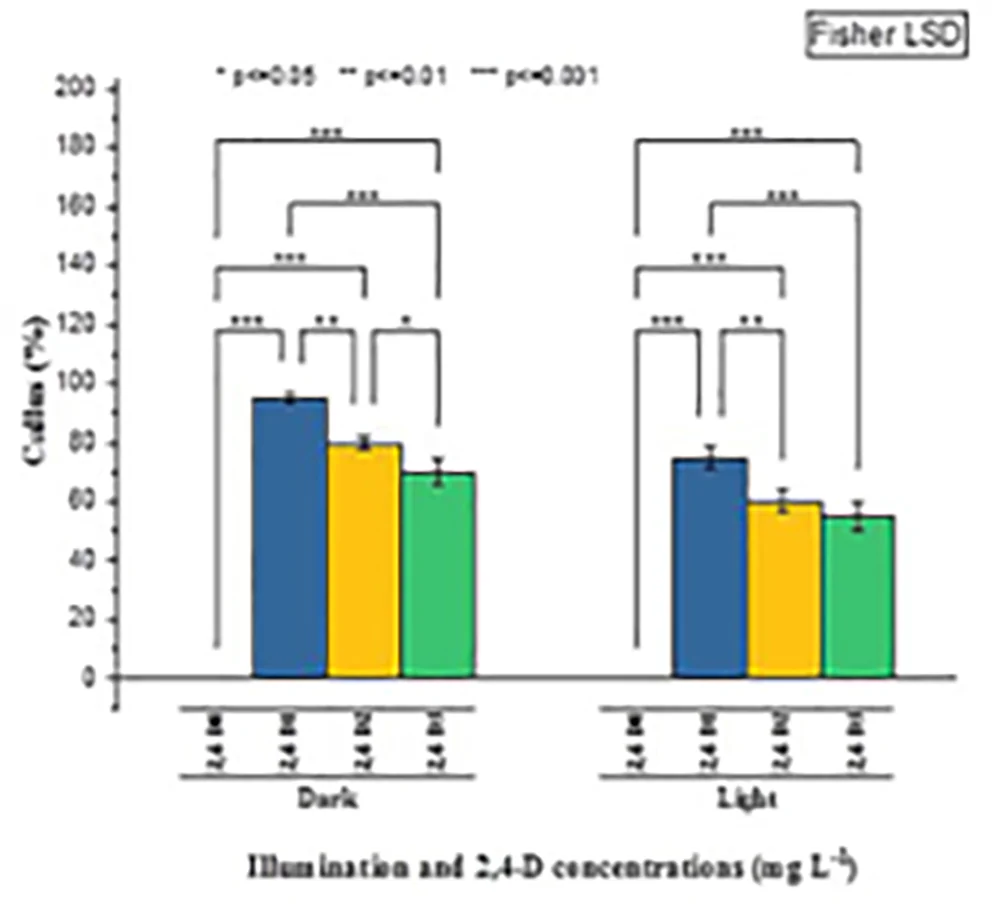
Effect of light conditions and 2,4-D concentrations on callus formation. X-axis (Abscissa): 2,4-D concentrations and illumination type: Dark and light, y-axis (ordinate): Callus percentage.

Statistical analysis, as shown in Figure 1 revealed that the growth regulator 2,4-D had a significant effect on callus induction. It was found that the optimal concentration for callus formation from leaf tissue was 1 mg L ^−1^ of 2,4-D added to the nutrient medium, which reached 95%, while an increase in concentration above this level led to a significant decrease in the rate of callus formation.

On the other hand, lighting had a relatively limited effect on callus induction compared to the effect of 2,4-D growth regulator concentration. The highest callus production values were recorded under complete darkness, and all auxin containing treatments outperformed their counterparts grown under lighting conditions ( Figure 1).

### Effect of illumination and 2,4-D concentrations on callus fresh weight (g)

**Figure 2.**
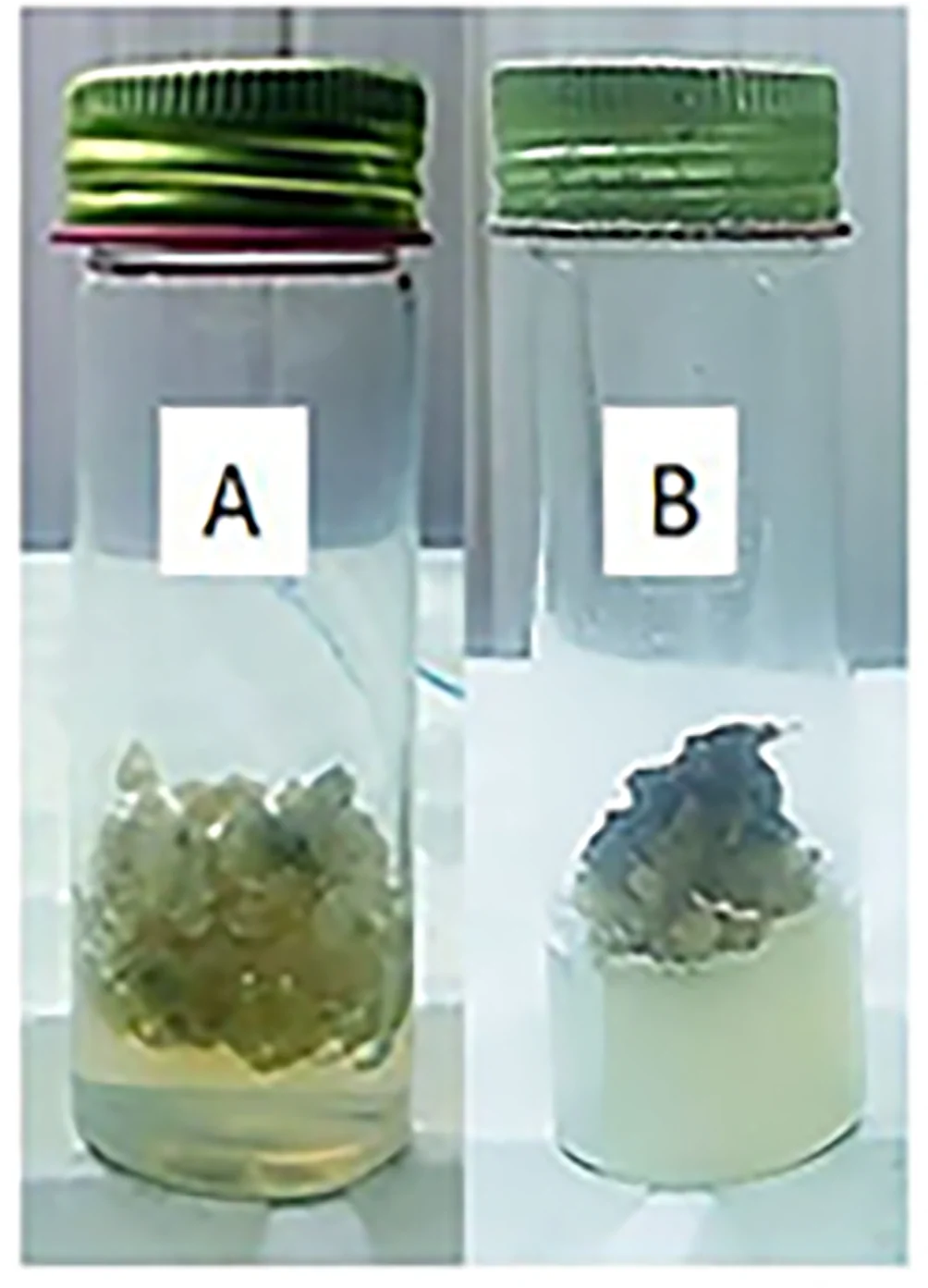
Effect of illumination (light (A) and dark (B)) on the growth of callus induced from lavender leaves, after four weeks.

The results of statistical analysis according to the data shown in Table 2 showed a statistically significant difference in the fresh weight values of callus induced from lavender leaf tissue, where the fresh weight of callus differed according to concentration (0, 1, 2 and 3) mg L ^−1^ of 2,4-D added to the MS nutrient medium. The auxin concentration of 1 mg L ^−1^ showed the highest significant value for callus fresh weight, which reached 0.796 g. An increase in auxin concentration above this level led to a gradual decrease in the rate of callus formation, indicating that high auxin concentrations have an inhibitory effect on the callus formation process. It should be noted that the auxin free treatment 0 concentration did not show any visible or statistical response in callus formation, confirming the pivotal role of auxin in initiating induction and stimulating cell division in plant leaf tissues. As for the effect of lighting, the results showed a clear superiority of callus growth under conditions of complete darkness for all concentrations studied, with the average fresh weight values under these conditions showing a remarkable superiority of 0.557 g compared to their counterparts grown under lighting conditions, which reached 0.527 g. The effect of the light factor can be observed in Figure 2, where the callus appeared yellowish brown, while in the dark, the callus appeared white yellowish. This difference in response may be due to the inhibitory effect of light on callus induction and growth processes. It can be concluded that the process of callus induction from lavender leaf tissue requires a delicate balance between the optimal concentration of auxin and suitable environmental conditions, emphasising the complex interaction between hormonal and environmental factors in regulating growth and differentiation processes in plant cells. The results also show that the highest fresh weight of callus was achieved at a concentration of 1 mg L ^−1^ of regulator 2,4-D when grown under dark conditions, reaching 0.830 g. While no value was recorded for fresh weight of callus (0 g) in treatments free of auxin under dark or light conditions. This confirms the importance of auxin as a basic factor in callus formation.

**Table 2.** Effect of illumination and 2,4-D concentrations on callus fresh weight (g).

Light conditions	2,4-D (mg L ^−1^)				
	0	1	2	3	Mean
**Dark**	0.000	0.830	0.752	0.647	0.557
**Light**	0.000	0.762	0.723	0.624	0.527
LSD _0.05_	0.014				0.007
**Mean**	0.000	0.796	0.737	0.635	
LSD _0.05_	0.010				

### Effect of illumination and 2,4-D on callus dry weight of callus (g)

Effect of auxin on dry weight of callus the results of statistical analysis shown in Table 3. Indicate a clear variation in biological response when treating lavender *Lavandula spp.* leaf tissues with different concentrations of the growth regulator 2,4-D. A concentration of 1 mg L ^−1^ of 2, 4-D recorded the highest significant dry weight of callus 0.064 g. An increase in concentration to 2 mg L ^−1^ resulted in a significant decrease in dry weight 0.058 g, while a concentration of 3 mg L ^−1^ recorded a decrease of 0.046 g. The auxin free treatment did not show any visible or statistical response in callus formation, confirming the pivotal role of auxin in the process of induction and initiation of cell division.

**Table 3.** Effect of light conditions and 2,4-D concentrations on callus dry weight (g).

Light conditions	2,4-D (mg L ^−1^)				Mean
	0	1	2	3	
**Dark**	0.000	0.068	0.059	0.048	0.044
**Light**	0.000	0.061	0.057	0.044	0.040
LSD _0.05_	0.002				0.001
**Mean**	0.000	0.064	0.058	0.046	
LSD _0.05_	0.001				

Statistical analysis showed a significant interaction between auxin concentration and lighting conditions. Treatments under lighting conditions recorded an average dry weight of 0.040 g, while treatments in complete darkness significantly exceeded this with an average dry weight of 0.044 g.

### Effect of 2,4-D and BA on fresh weight of callus (g)

The results in Table 4, Showed that adding synthetic auxin of type 2,4-D at different concentrations to the nutrient medium had a significant statistically significant effect on the fresh weight of the callus formed. A clear response to the concentrations added to the nutrient medium was observed, with the low concentration 1 mg L ^−1^ outperforming all other treatments, recording the highest significant value for fresh weight of 0.920 g. With an increase in hormone concentration to 2 and then 3 mg L ^−1^, a gradual and significant decline in growth efficiency was observed, with the fresh weight of callus decreasing to 0.811 g and 0.632 g, respectively. This decrease indicates the possibility of an inhibitory effect of high concentrations of auxin on the process of development and growth.

**Table 4.** Effect of 2,4-D and BA on fresh weight (g) of callus.

BA (mg L ^−1^)	2,4-D (mg. L ^−1^)			Mean
	1	2	3	
0	0.838	0.728	0.628	0.731
1	0.880	0.843	0.636	0.786
2	1.042	0.863	0.634	0.846
LSD _0.05_	0.018			0.010
**Mean**	0.920	0.811	0.632	
LSD _0.05_	0.010			

On the other hand, the results also showed statistically significant differences between the different concentrations of cytokinin benzyladenine (BA) added to the nutrient medium. The effect of BA was characterised by a positive linear relationship, where the growth rate of callus measured by fresh weight increased significantly with increasing hormone concentration. Growth peaked at a concentration of 2 mg L ^−1^, achieving the highest fresh weight value of 0.846 g. In contrast, the treatment containing a concentration of 0 mg L ^−1^ free of cytokinin recorded the lowest fresh weight value of 0.731 g, highlighting the vital importance of cytokinin in supporting cell division and callus formation. The most significant scientific importance of this study lies in evaluating the complementary and reciprocal effects of these two hormones when used together. The results showed a clear superiority of some combinations over others. The addition of a combination of auxin 2,4-D at a concentration of 1 mg L ^−1^ in conjunction with cytokinin BA at a concentration of 2 mg L ^−1^ resulted in the highest total value of 1.041 g. This result reflects a synergistic effect between the two hormones, each enhancing the effect of the other, leading to optimal stimulation of growth and development processes. In contrast, the combination of auxin 2,4-D at a concentration of 3 mg. L ^−1^ with cytokinin at a concentration of 0 mg. L ^−1^ recorded the lowest fresh weight value, which was 0.628 g. This poor performance confirms the negative effects of high auxin concentrations in the absence of a balancing factor such as cytokinin, supporting the hypothesis that the balance between these two hormones, rather than the presence of only one, is the decisive factor in directing plant cell growth and differentiation in tissue cultures.

### Effect of 2,4-D and BA on dry weight of callus (g)

The results in Table 5 showed a clear response when auxin was added at different concentrations to the nutrient medium. The concentration of 1 mg L ^−1^ significantly outperformed all other treatments, recording the highest dry weight of callus at 0.064 g. This result indicates that this concentration is optimal for stimulating cell division and hypertrophy, which positively affects biomass accumulation. However, a gradual and significant decline in growth efficiency was observed with increasing auxin concentration. Dry weight decreased to 0.060 g at a concentration of 2 mg L ^−1^ and to 0.044 g at a concentration of 3 mg L ^−1^. This decrease indicates that higher concentrations of auxin may have an inhibitory or toxic effect on cells, hindering their growth and limiting callus formation, which is common in plant responses to growth regulators when the optimum limit is exceeded.

**Table 5.** Effect of 2,4-D and BA on dry weight (g) of callus.

BA (mg L ^−1^)	2,4-D (mg L ^−1^)			Mean
	1	2	3	
**0**	0.065	0.055	0.041	0.054
**1**	0.062	0.060	0.042	0.055
**2**	0.070	0.065	0.050	0.062
L.S.D _0.05_	0.002			0.001
**Mean**	0.065	0.060	0.044	
L.S.D _0.05_	0.001			

The results showed statistically significant differences in the dry weight of callus when different concentrations of cytokinin were added. The effect of cytokinin was characterised by a positive linear relationship, with the dry weight of induced callus increasing significantly with increasing hormone concentration. This effect peaked at a concentration of 2 mg L ^−1^, achieving the highest dry weight value of 0.062 g. This confirms the vital role of cytokinin in regulating cell division and differentiation, which is a complementary and important factor in the callus induction process.

The greatest scientific value of this study lies in assessing the combined and complementary effects of auxin and cytokinin hormones, as it is known that the balance between them is the main driver of plant cell growth. In this context, the results showed a significant advantage for the combination of auxin at a concentration of 1 mg. L ^−1^ with cytokinin at a concentration of 2 mg. L ^−1^. This combination achieved the highest dry weight value for callus 0.070 g. This outstanding performance is attributed to the synergistic effect between the two hormones, where auxin at its optimal concentration 1 mg. L ^−1^ reduces cell elongation, while cytokinin at its optimal concentration 2 mg L ^−1^ promotes cell division and prevents differentiation, which together leads to optimal cell mass enlargement and higher dry matter accumulation.

In contrast, the combination with the highest concentration of auxin 3 mg. L ^−1^ and a complete absence of cytokinin 0 mg L ^−1^ recorded the lowest dry weight value, at 0.041 g. These results confirm that the success of callus induction and its maximum growth measured by dry weight depend critically on the selection of the optimal concentration of each plant regulator separately and more importantly, on the selection of the optimal combination of the two. The interaction between auxin and cytokinin is what directs the final physiological response of plant tissues.

### Effect of nutrient medium components and chitosan on callus fresh weight (g)

The results of statistical analysis, shown in Table 6, revealed a clear variation in the components of different nutrient media in stimulating callus growth. The comparison showed that the nutrient medium B5 significantly outperformed the other media, recording the highest average fresh weight of callus induced from leaf tissue, with a value of 2.197 g. In contrast, the MS medium showed the lowest fresh weight growth rate for the same type of callus, with a value not exceeding 1.233 g.

**Table 6.** Effect of nutrient medium components and chitosan on callus fresh weight(g).

Medium components	chitosan (mg L ^−1^)				Mean
	0	50	100	200	
**MS**	1.000	1.206	1.904	0.824	1.233
**B5**	1.892	2.374	2.392	2.131	2.197
L.S.D _0.05_	0.060				0.030
**Mean**	1.446	1.790	2.148	1.477	
L.S.D _0.05_	0.042				

**Figure 3.**
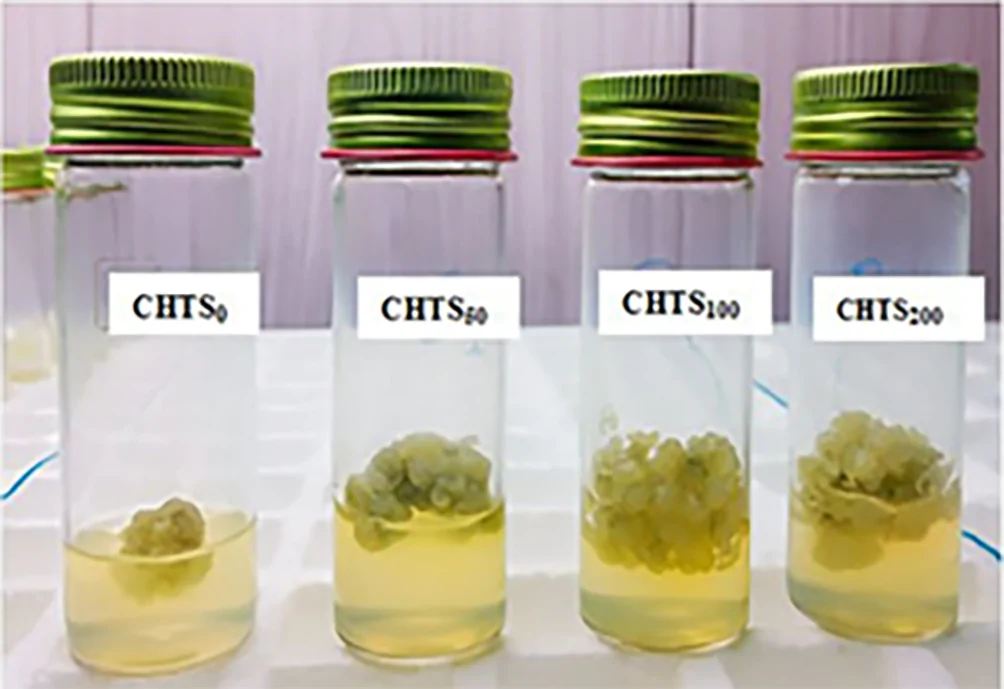
Effect of chitosan concentrations: CHTS _0_: without chitosan, CHTS _50_: 50 mg chitosan per liter, CHTS _100_:100 mg chitosan per liter and CHTS _200_: 200 mg chitosan per liter on callus growth of lavender leaves, after four weeks.

As for the results related to the analysis of the effect of chitosan concentration, presented in Table 6. They showed statistically significant differences between the four concentrations studied (0, 50, 100 and 200) mg L ^−1^ was observed that all tested concentrations of chitosan significantly outperformed the control group ( Figure 3), confirming the positive effect of this substance in stimulating soft weight gain in callus. This effect followed a clear pattern of proportionality, as the rate of soft weight gain in calves increased significantly with increasing chitosan concentration, reaching its highest value at a concentration of 100 mg L ^−1^, with a peak of 2.148 g. Performance reversed significantly when the highest concentration 200 mg L ^−1^, where the fresh weight of the callus decreased compared to the optimal concentration 100 mg L ^−1^, indicating that the positive effect of chitosan is at its maximum at a specific concentration, and that increasing the concentration beyond this limit may lead to adverse results.

### Effect of nutrient medium components and chitosan on dry weight of callus (g)

The results of statistical analysis, as shown in Table 7, revealed a clear variation in the performance of nutrient medium components in terms of their effect on the dry weight of callus. The results showed a marked superiority of the B5 diet in achieving the highest average dry weight of callus induced from leaf tissue, recording a value of 0.103 g. In contrast, the nutrient medium MS recorded the lowest dry weight, with a value of 0.076 g. indicating that the composition of the nutrient medium B5 was more suitable and effective in promoting growth and dry matter accumulation in callus compared to the medium MS.

**Table 7.** Effect of nutrient medium components and chitosan on dry weight of callus (g).

Medium components	Chitosan (mg L ^−1^)				Mean
	0	50	100	200	
**MS**	0.068	0.071	0.087	0.081	0.076
**B5**	0.101	0.103	0.106	0.102	0.103
L.S.D _0.05_	0.002				0.001
**Mean**	0.084	0.087	0.096	0.091	
L.S.D _0.05_	0.001				

As for the results shown in Table 7, Statistical analysis revealed statistically significant differences between the different concentrations of chitosan included in the study (0, 50, 100 and 200) mg L ^−1^. It was observed that all concentrations treated with chitosan significantly outperformed the control group, to which no concentration of the substance was added 0 mg L ^−1^. This superiority confirms the stimulating and positive effect of chitosan on callus growth.

The results also revealed a positive correlation between increased chitosan concentration and the dry weight of induced callus, with values increasing steadily with higher concentrations, peaking at a concentration of 100 mg L ^−1^. The average dry weight of the callus reached its highest value, amounting to 0.096 g. A decline in this positive effect was also observed when using the highest concentration 200 mg L ^−1^, where the average dry weight decreased to 0.091 g. This decrease indicates that the stimulating effect of chitosan follows an optimum pattern, achieving maximum growth response, and that exceeding this concentration may have an inhibitory or less effective effect on the accumulation of dry matter in callus.

## Discussion

Growth regulators, especially auxins, are key factors in stimulating callus formation. The addition of auxin to the nutrient medium elicited a response in the leaf tissues of lavender plants, stimulating callus formation. The added auxin induces fundamental changes in the plant cells in contact with the medium and stimulates the initiation of cell division. During this stimulation phase, constructive processes such as protein synthesis and DNA replication are activated, followed by a series of changes in cellular activity that culminate in cell division and the formation of a callus mass covering most parts of the plant fragment.

The response of plant tissue varies depending on the concentration of auxin in the nutrient medium. An increase in concentration promotes callus formation until the optimum concentration of 1 mg. L ^−1^ is reached, which achieved the highest callus formation rate 95%, in addition to an increase in fresh and dry weight. Conversely, exceeding this optimal concentration, as observed at concentrations of 2 and 3 mg. L ^−1^, leads to adverse effects that inhibit growth. The experiment also showed that light was an inhibitory factor for callus growth induced from lavender leaves compared to dark conditions, which were more conducive to growth. The reason for the reduced callus growth under light can be attributed to the reduced effectiveness of auxin.

It is important to note that continuing to supplement the nutrient medium with auxin at the optimal concentration in order to increase callus production may inhibit growth due to auxin accumulation, necessitating the addition of cytokinin. Cytokinin plays an important role in sustaining callus growth. and previous studies indicate that the interaction between auxins and cytokinins at different concentrations is a decisive factor in achieving optimal hormonal balance ( Jabr and Mohsen, 2024; Tilkat and Onay, 2009). In this regard, the treatment consisting of 1 mg L ^−1^ auxin and 2 mg L ^−1^ benzyladenine BA recorded the highest fresh and dry weight values, indicating that it is a suitable combination for sustained callus growth. This can be explained by the ability of cytokinins, especially benzyladenine, to promote nutrient uptake, stimulate cell division and inhibit protein degradation, which supports the division process when the appropriate hormonal balance is achieved. Cytokinins also promote the synthesis of RNA, proteins, and enzymes within the cell. Overall, the increase in soft and dry weight of callus reflects changes in cellular components such as carbohydrates, proteins and amino acids associated with the process of division and growth ( Bulya *et al.*, 2023). Therefore, the balance between auxins and cytokinins in the nutrient medium remains a key determinant of continued cell division and callus growth. In addition to growth regulators, the type of nutrient medium played an important role in fresh and dry weight increase, with nutrient medium B5 outperforming MS medium, which is attributed to the difference in the composition of the two media.

Bioactive chitosan was also used as a callus growth stimulator, and the results were consistent with previous studies on the role of chitosan in stimulating growth ( Pirbalouti *et al*., 2017; Byczyńska, 2018; El-Khateeb *et al*., 2018; Atteya *et al*., 2023). chitosan treatments recorded significant increases in growth indicators and a positive correlation was observed between chitosan concentration up to a certain level and callus growth, with the highest fresh and dry weight values recorded at a concentration of 100 mg. L ^−1^. This stimulation is attributed to the biological properties of chitosan, which enhances the absorption of key nutrients such as nitrogen and phosphorus, thereby supporting metabolic processes and the accumulation of organic matter. This was reflected in an increase in fresh weight due to higher water and organic matter content and dry weight due to the accumulation of proteins and carbohydrates, resulting in improved biomass of the callus. Stimulation must be limited and sensitive to concentration; at a concentration of 200 mg L ^−1^, growth indicators decreased significantly, and this decrease can be explained physiologically by ionic disturbance and inhibition of nutrient absorption.

## Conclusion

This study evaluated the effects different concentrations of 2,4-D have on tissue culture callus induction of lavender leaf explants. From the results, all of the hormone concentrations caused callus formation to begin, but the MS medium with 1 mg L ^−1^ of 2,4-D was the most efficient and most effective in terms of the rate of callus formation. The addition of the growth regulator combination 2,4-D with BA. Significantly improved the capacity of callus formation. it was evident that the synergistic effect between the two hormone groups was responsible for the significant increase in callus yield. With respect to the biostimulant chitosan, it was concluded that chitosan addition to the culture medium enhanced the growth of the formed callus.

## Data Availability

All raw data files underlying the results of this study are available on OSF: **Raw data for lavender study**, https://doi.org/10.17605/OSF.IO/AMV4Z ( Almehemdi 2025). This project contains the following underlying data:
• Lavenderdata1.xlsx Data are available under the terms of the Creative Commons Attribution 4.0 International license (CC-BY 4.0).
