# Supplementary material for: Evaluation of the efficiency of the optimal combination of  hormonal, physical  and biological stimuli in the induction and growth of callus in Lavandula angustifolia Mill
Source: F1000Res. 2026 Jul 13;15:407. Originally published 2026 Mar 18. [Version 3] doi: 10.12688/f1000research.173637.3 (PMC13550908; doi:10.12688/f1000research.173637.3)
Supplement: Peer review history

# Reviewer report: Reviewer response for version 3

**DOI:** 10.5256/f1000research.205652.r500911

**Published:** 2026-07-22

**Authors:** Duha Mysire Majeed

**Affiliations:** 1 Plant Biotechnology, Al-Nahrain University / Biotechnology Research center, Baghdad, Iraq

**I have reviewed the revised version and the authors have adequately addressed my comments. I have no further comments.**

Is the work clearly and accurately presented and does it cite the current literature?

Partly

If applicable, is the statistical analysis and its interpretation appropriate?

Yes

Are all the source data underlying the results available to ensure full reproducibility?

Yes

Is the study design appropriate and is the work technically sound?

Partly

Are the conclusions drawn adequately supported by the results?

Partly

Are sufficient details of methods and analysis provided to allow replication by others?

Partly

Reviewer Expertise:

Plant Biotechnology, Plant Tissue Culture, Molecular Biology, Plant Genetic Engineering

I confirm that I have read this submission and believe that I have an appropriate level of expertise to confirm that it is of an acceptable scientific standard.

# Reviewer report: Reviewer response for version 2

**DOI:** 10.5256/f1000research.202213.r493248

**Published:** 2026-07-06

**Authors:** Tassanai Jaruwattanaphan

**Affiliations:** 1 Kasetsart University, Bangkok, Thailand

The authors answered well to the concerns I addressed in my earlier review on Version 1. The taxonomic inconsistency has been resolved, the Y-axis scaling error in figure 1 has been corrected, chitosan characterization has been added, the use of GA₃ has been clarified, a helpful workflow diagram has been included, and the terminology has been revised from “synergistic” to “optimal combination”, all of which have significantly improved the clarity and consistency of the manuscript.

 I would like to draw the authors' attention to two points from my previous report where the response letter describes a revision that I could not locate in the current text. It is possible these were simply missed during the final edit, so I mention them for the authors' convenience:

- Auxin mechanism (previous Comment 13). The Discussion still reads that "the reduced callus growth under light can be attributed to the reduced effectiveness of auxin." I'd suggest either adding a supporting reference or slightly rewording this sentence to reflect a more precise physiological mechanism.
- B5 medium superiority (previous Comment 14). The Discussion still attributes B5's better performance generally to "the difference in the composition of the two media." A brief note on which specific components (e.g., nitrogen source ratio, vitamins) likely drive this difference, with a supporting citation, would strengthen this section. I noticed another reviewer of this version raised a similar observation, so this may simply need one more pass.

I'd also appreciate confirmation that F-values and p-values were included in Tables 2–5 as described in the response letter, as this wasn't fully clear to me from the version I reviewed.

Is the work clearly and accurately presented and does it cite the current literature?

Partly

If applicable, is the statistical analysis and its interpretation appropriate?

Partly

Are all the source data underlying the results available to ensure full reproducibility?

Yes

Is the study design appropriate and is the work technically sound?

Partly

Are the conclusions drawn adequately supported by the results?

Partly

Are sufficient details of methods and analysis provided to allow replication by others?

Partly

Reviewer Expertise:

Plant germplasm conservation, plant molecular genetics, plant tissue culture, and cytogenetics.

I confirm that I have read this submission and believe that I have an appropriate level of expertise to confirm that it is of an acceptable scientific standard, however I have significant reservations, as outlined above.

# Response

**Published:** 2026-07-08

**Authors:** MOHAMMED ABDULGAFOR

**Affiliations:** biotechnology, University of Anbar, Ramadi, Al Anbar Governorate, Iraq

We sincerely thank the reviewer for the careful re-evaluation of our revised manuscript and for the positive assessment of the improvements made. We greatly appreciate your recognition that the taxonomic inconsistency, Figure 1 scaling, chitosan characterization, clarification of GA₃ application, inclusion of the workflow diagram, and the revised terminology have substantially improved the quality and clarity of the manuscript.

 We also thank you for pointing out the remaining issues that were unintentionally overlooked during the final revision.

 Response to Comment 13 (Auxin mechanism):

 We have revised the Discussion to provide a more accurate explanation of the physiological mechanism underlying the reduced callus growth under light conditions. The statement has been reworded to avoid oversimplification, and an appropriate supporting reference has been added to strengthen this interpretation.

 Response to Comment 14 (B5 medium superiority):

 We agree with the reviewer's suggestion. The Discussion has been expanded to explain that the superior performance of B5 medium is likely associated with differences in nutrient composition, particularly the nitrogen source ratio and vitamin formulation, compared with MS medium. Relevant supporting references have also been incorporated to support this explanation.

 F-values and p-values in Tables 2–5:

 Thank you for requesting clarification. We confirm that the F-values and corresponding p-values have now been clearly presented in Tables 2–5 as indicated in our response letter. We carefully reviewed the final version to ensure that these statistical values are included and correctly displayed.

 We sincerely appreciate your constructive comments and careful review. Your valuable suggestions have helped us further improve the scientific quality, clarity, and accuracy of the manuscript.

# Reviewer report: Reviewer response for version 2

**DOI:** 10.5256/f1000research.202213.r494919

**Published:** 2026-07-01

**Authors:** Huda Enaya Mahood

**Affiliations:** 1 University of Al-Qadisiyah, Al Diwaniyah, Iraq

The manuscript addresses an interesting topic with potential value for plant tissue culture research. However, several sections require substantial revision.

1. The introduction does not fully match the study title. The role of physical stimuli is not sufficiently discussed, despite being a key component of the research. Please include a brief background on physical stimuli and clearly align the study objective with the title.
2. The Materials and Methods section is generally well-organized, but some scientific and linguistic points need correction.

The details of the culture medium are lengthy and unnecessary (such as transferring the solution to the graduated cylinder and boiling at 100°C). These could be shortened, and the focus could be on the essential information.

 Sterilization: The phrase "according to the method used by the researcher" is inappropriate. It would be better to state that it was performed according to a published protocol or to describe the method directly.

 The experimental design is good, but the number of replicates per experiment and the number of plant parts (explants) used per replicate should be specified.

 Results: This section is the weakest part of the manuscript, not because the results are bad, but because the way they are presented and discussed needs significant improvement.  

 Discussion

 The numerical results are repeated; the discussion should focus on interpretation rather than mere presentation.

 The discussion of the effect of darkness (physical stimulus) is too brief, even though the title focuses on physical stimuli. It should be expanded and linked to previous studies.

 The reason for the superiority of B5 over MS was not adequately discussed; the authors simply stated the general phrase "due to the difference in composition." It would be better to clarify which components of B5 might be responsible for this and cite references.

 (The reason for the reduced callus growth under light can be attributed to the reduced effectiveness of auxin). This statement needs a reference or rewording.

 The conclusion is clear, but it mainly summarizes the results. Please strengthen it by highlighting the main significance of the study and ensuring that it reflects all aspects of the work, including the effect of the physical and biological stimuli.

Is the work clearly and accurately presented and does it cite the current literature?

Partly

If applicable, is the statistical analysis and its interpretation appropriate?

Yes

Are all the source data underlying the results available to ensure full reproducibility?

Yes

Is the study design appropriate and is the work technically sound?

Yes

Are the conclusions drawn adequately supported by the results?

Partly

Are sufficient details of methods and analysis provided to allow replication by others?

Partly

Reviewer Expertise:

Plant Biotechnology

I confirm that I have read this submission and believe that I have an appropriate level of expertise to confirm that it is of an acceptable scientific standard, however I have significant reservations, as outlined above.

# Response

**Published:** 2026-07-05

**Authors:** MOHAMMED ABDULGAFOR

**Affiliations:** biotechnology, University of Anbar, Ramadi, Al Anbar Governorate, Iraq

**Response to Reviewer**

 We sincerely thank the reviewer for the careful evaluation of our manuscript and for the constructive comments, which have significantly improved its scientific quality. All comments were carefully considered, and the manuscript has been revised accordingly. Our point-by-point responses are presented below.

**Comment 1:** *The introduction does not fully match the study title. The role of physical stimuli is not sufficiently discussed, despite being a key component of the research. Please include a brief background on physical stimuli and clearly align the study objective with the title.*

**Response:** Thank you for this valuable suggestion. The Introduction has been revised to include a dedicated background describing the role of physical stimuli, particularly light and darkness, in regulating callus induction and growth through their influence on endogenous hormones, cellular dedifferentiation, and morphogenesis. In addition, the study objective has been rewritten to better reflect the title by emphasizing the integrated evaluation of hormonal, physical, and biological stimuli.

**Comment 2:** *The details of the culture medium are lengthy and unnecessary.*

**Response:** We agree with the reviewer. The description of culture medium preparation has been shortened by removing unnecessary procedural details while retaining all essential information required for reproducibility.

**Comment 3:** *Sterilization: The phrase "according to the method used by the researcher" is inappropriate.*

**Response:** Thank you for pointing this out. The sterilization procedure has been revised by removing this phrase and replacing it with a clear description of the protocol used, together with the appropriate reference.

**Comment 4:** *The number of replicates and explants per replicate should be specified.*

**Response:** This information has now been added to the Materials and Methods section. The number of biological replicates and the number of explants used in each replicate are clearly stated.

**Comment 5:** *The Results section is weak because of the way the results are presented and discussed.*

**Response:** We appreciate this important observation. The Results and Discussion have been extensively revised to improve clarity, organization, and scientific interpretation. The discussion now emphasizes the biological significance of the findings rather than simply describing the numerical results.

**Comment 6:** *The numerical results are repeated; the discussion should focus on interpretation.*

**Response:** We agree. Repetitive descriptions of numerical values have been removed, and the Discussion has been rewritten to focus on interpreting the results in relation to previous studies and the underlying physiological mechanisms.

**Comment 7:** *The discussion of the effect of darkness is too brief.*

**Response:** Thank you for this suggestion. The discussion concerning the influence of darkness has been substantially expanded. It now explains the physiological basis of darkness-induced callus formation, including its effects on auxin stability, cellular dedifferentiation, and morphogenic responses, and is supported by additional relevant literature.

**Comment 8:** *The superiority of B5 over MS was not adequately discussed.*

**Response:** We appreciate this comment. The Discussion has been expanded to explain the likely reasons for the superior performance of Gamborg's B5 medium, including differences in nitrogen sources, nitrate/ammonium balance, vitamin composition, and their influence on cellular metabolism and biomass accumulation. Appropriate references have also been added.

**Comment 9:** *The statement regarding reduced auxin effectiveness under light requires a reference or rewording.*

**Response:** Thank you for identifying this issue. The statement has been revised and is now supported with appropriate references describing the influence of light on auxin metabolism and signaling.

**Comment 10:** *The conclusion mainly summarizes the results.*

**Response:** We agree with the reviewer. The Conclusion has been rewritten to emphasize the broader scientific significance of the study, highlighting the importance of integrating hormonal, physical, nutritional, and biological stimuli for optimizing callus production in *Lavandula angustifolia* and its potential applications in plant biotechnology and secondary metabolite production.

 Once again, we sincerely thank the reviewer for the insightful comments and valuable suggestions, which have substantially improved the quality, clarity, and scientific rigor of the manuscript.

# Reviewer report: Reviewer response for version 2

**DOI:** 10.5256/f1000research.202213.r493247

**Published:** 2026-06-29

**Authors:** Duha Mysire Majeed

**Affiliations:** 1 Plant Biotechnology, Al-Nahrain University / Biotechnology Research center, Baghdad, Iraq

The authors have satisfactorily addressed the concerns raised in my previous review. The manuscript has been improved in terms of methodology description, statistical analysis, discussion, and overall presentation. I consider the revised version suitable for indexing.

Is the work clearly and accurately presented and does it cite the current literature?

Partly

If applicable, is the statistical analysis and its interpretation appropriate?

Yes

Are all the source data underlying the results available to ensure full reproducibility?

Yes

Is the study design appropriate and is the work technically sound?

Partly

Are the conclusions drawn adequately supported by the results?

Partly

Are sufficient details of methods and analysis provided to allow replication by others?

Partly

Reviewer Expertise:

Plant Biotechnology, Plant Tissue Culture, Molecular Biology, Plant Genetic Engineering

I confirm that I have read this submission and believe that I have an appropriate level of expertise to confirm that it is of an acceptable scientific standard.

# Reviewer report: Reviewer response for version 1

**DOI:** 10.5256/f1000research.191471.r476658

**Published:** 2026-05-08

**Authors:** Tassanai Jaruwattanaphan

**Affiliations:** 1 Kasetsart University, Bangkok, Thailand

This manuscript investigates the effects of 2,4-D, BA, light conditions, culture medium (MS versus B5), and chitosan on callus induction and growth in lavender. The main topic of the study is relevant to the field of plant biotechnology, and the presented results, especially positive effects of 1 mg L⁻¹ 2,4-D in dark conditions, along with 2 mg L⁻¹ BA, B5 medium, and 100 mg L⁻¹ chitosan on callus production, are considered acceptable. However, the manuscript needs major revision to be considered scientifically accurate and acceptable for indexing.

 Is the study design appropriate and is the work technically sound? **Partly**

- The three experimental stages are not clearly related as the settings adjusted in phase 1 were not explicitly taken to phases 2 and 3. Thus, it is impossible to evaluate the alleged synergistic effects. The explant description is also not detailed enough, as key characteristics such as leaf age, location and size are not included.
- Furthermore, the callus development experiment lacks a zero-auxin control and the chitosan experiment does not state the plant growth regulators in the basal media.
- The manuscript refers to *Lavandula angustifolia* Mill. in the Introduction but uses *Lavandula* spp. in the Results. This inconsistency is taxonomically unacceptable and must be corrected throughout.
- The authors could define sequential experimental design and possibly include a basic workflow diagram illustrating how each phase was related.

Are sufficient details of the methods and analysis provided to allow replication by others? **Partly**

- Methodological information is partially adequate for reproduction. Basic culture conditions are presented; however, numerous important aspects are omitted.
- Inadequate description of explant preparation (leaf age, location, size and developmental stage are not provided)
- There is no characterization of chitosan; degree of deacetylation (DD%) and molecular weight should be given since they have a substantial influence on biological activity.
- While the sterilizing approach (3% NaOCl for 15 min) may be too severe, there is no information on contamination or explant survival rates. No mention of strategies to reduce oxidative browning either.
- The use of GA₃ in germination media is not supported or referred to; however, it may affect explant physiology and subsequent induction of callus.
- The physiological condition and origin of the 150 mg callus inoculum employed in later phases, such as treatment source, callus age, and subculture history, are unknown.

Is the statistical analysis and its interpretation appropriate? **Partly**

- The statistical approach (CRD analyzed by ANOVA with LSD at p ≤ 0.05) is generally appropriate, and the use of ten replicates per treatment is commendable.
- However, ANOVA outputs are not reported. Tables include only treatment means and LSD₀.₀₅ values, without F-values or p-values for main effects and interactions.
- Pairwise comparison letters are absent from all tables, making treatment differences difficult to interpret.
- Figure 1 contains a major scaling error, as the Y-axis exceeds 100% despite representing callus formation percentage.
- Error bars are not defined (SD or SE), and the large variation observed in some treatments is not discussed.

Are the conclusions drawn adequately supported by the results? **Partly**

- Overall, indicated trends are generally consistent with provided data; however, several findings are misleading or inadequately supported.
- The notion of “synergistic efficiency” has no justification. Most factors were tested in isolation in the studies, and no real cross-factor interaction analysis was performed.
- Physiologically, the concept of auxin action is incorrect. Auxin is mostly related to cell division and dedifferentiation in callus induction, not to decreased cell elongation.
- The reason given for the advantage of B5 medium is too superficial and should mention precise compositional differences between B5 and MS media.
- The introduction highlights the synthesis of secondary metabolites, but no metabolite analysis was done; hence, the data do not support these assertions.
- Only biomass measurements were taken to determine callus quality, and essential features such as texture, color, viability, and oxidative browning were not investigated.

**Comments on Figures**

- Figure 1 contains a critical Y-axis error (scale to 200%) that must be corrected. Error bar definitions must be stated, and the source of high variability acknowledged.
- Figure 2 is of insufficient quality for publication. The image is low resolution, includes distracting background elements, does not specify which 2,4-D treatment is shown, and lacks a scale bar.
- Figure 3 does not specify whether images are from MS or B5 medium.
- No photographic documentation is provided for the 2,4-D + BA experiment.

Is the work clearly and accurately presented and does it cite the current literature?

Partly

If applicable, is the statistical analysis and its interpretation appropriate?

Partly

Are all the source data underlying the results available to ensure full reproducibility?

Yes

Is the study design appropriate and is the work technically sound?

Partly

Are the conclusions drawn adequately supported by the results?

Partly

Are sufficient details of methods and analysis provided to allow replication by others?

Partly

Reviewer Expertise:

Plant germplasm conservation, plant molecular genetics, plant tissue culture, and cytogenetics.

I confirm that I have read this submission and believe that I have an appropriate level of expertise to state that I do not consider it to be of an acceptable scientific standard, for reasons outlined above.

# Response

**Published:** 2026-05-21

**Authors:** MOHAMMED ABDULGAFOR

**Affiliations:** biotechnology, University of Anbar, Ramadi, Al Anbar Governorate, Iraq

**Response to the Reviewer**

**Authors’ Response to the First Reviewer’s Report (Tassanai Jaruwattanaphan)**

 We would like to express our sincere appreciation to the esteemed reviewer for the valuable time devoted to the careful evaluation of our manuscript, as well as for the constructive and insightful comments provided. The reviewer’s detailed observations have been highly valuable in improving the scientific quality, clarity, and overall rigor of the manuscript.

 In response to the reviewer’s suggestions and recommendations, we have thoroughly revised the manuscript and carefully addressed all comments raised. Below, we provide a detailed point-by-point response outlining the revisions made accordingly.

**First: Study Design and Technical Soundness**

**Comment 1:** *The relationship between the three experimental phases was unclear, making it difficult to assess the synergistic effect.*

**Response:**

 We sincerely appreciate the reviewer’s insightful comment regarding the clarity of the experimental design. In response, we have revised the Materials and Methods section by incorporating a new subsection entitled Sequential Experimental Design to better clarify the relationship among the three experimental phases and the rationale underlying their progression.

 Specifically, the study was structured in a hierarchically linked and sequential manner. **Phase 1** aimed to identify the optimal concentration of **2,4-D** under light conditions. Based on the outcomes of this phase, the most effective concentration ( **1 mg/L 2,4-D**) was selected and subsequently fixed for the following experiments conducted under dark conditions. This sequential approach was intentionally designed to ensure experimental consistency and to facilitate a clearer evaluation of the cumulative and synergistic effects among the study variables.

 In **second stage**, the induced callus obtained from explants cultured on Murashige and Skoog (MS) medium supplemented with 1 mg/L 2,4-D under dark conditions was subsequently transferred to media containing different combinations of auxin and cytokinin (BA) to determine the optimal hormonal combination for callus growth and maintenance.

 In **third stage**, the callus induced under the previously optimized hormonal conditions ( **1 mg/L 2,4-D + 2 mg/L BA**) was transferred to experimental media in which this hormonal combination was maintained as a fixed parameter. This phase was specifically designed to evaluate and compare the effects of different nutrient media and varying concentrations of **chitosan** added to the culture medium.

 To further improve the clarity of the experimental workflow and the interrelationship among the study phases, we have incorporated a workflow diagram (Figure 1) into the revised manuscript. This newly added figure visually illustrates the sequential progression of the experimental stages and the transitions between them, thereby facilitating a clearer understanding of the integrated and synergistic evaluation of the investigated factors

**Comment 2:** *The description of the leaflet is not sufficiently detailed (leaf age, location, size).*

**Response:**

 We sincerely thank the reviewer for this valuable and insightful observation. In response, we have substantially expanded the description of the explant source in the Materials and Methods section to provide greater experimental precision and reproducibility.

**Comment 3:** *The absence of an auxin-free control treatment in the callus development experiment.*

**Response:**

 We sincerely thank the reviewer for this important observation. The primary objective of the experiment was to evaluate callus proliferation and maintenance, rather than callus induction. During the preliminary callus induction phase (Phase 1), we observed that explants cultured on auxin-free medium failed to initiate callus formation, indicating that auxin supplementation was essential for successful callus induction in this species.

 Furthermore, an auxin-free   medium, combined with the addition of cytokinin, leads to the formation of vegetative shoots; for this reason, the (0 auxin) treatment was excluded from the study. 

**Comment 4:** *The growth regulators used in the base media for the chitosan experiment were not specified.*

**Response:**

 We appreciate the reviewer for highlighting this point. In response, we have revised the **Materials and Methods** section to explicitly specify the plant growth regulator composition used during the chitosan experiment.

**Comment 5:** *The taxonomic discrepancy between* ***Lavandula angustifolia*** *in the Introduction and* ***Lavandula spp.*** *in the Results.*

**Response:**

 We sincerely thank the reviewer for identifying this inconsistency. We fully acknowledge that maintaining taxonomic accuracy and consistency throughout the manuscript is essential. In response, the scientific nomenclature has been carefully reviewed and standardized across all sections of the manuscript. The species name has been consistently corrected to *Lavandula angustifolia* **Mill.**, which is the actual species utilized in all experimental procedures.

**Second: Methods and Replication Details**

**Comment 6:** *The characterization of chitosan is missing (degree of deacetylation and molecular weight).*

**Response:**

 We sincerely appreciate the reviewer for highlighting this important methodological detail. In response, the physicochemical characteristics of the chitosan used in the study have now been explicitly included in the Materials and Methods section to enhance methodological transparency and reproducibility.

**Comment 7:** *The sterilization protocol was harsh (3% NaOCl for 15 min), and no information was provided regarding contamination or tissue browning.*

**Response:**

 We sincerely thank the reviewer for this important observation. We acknowledge that the sterilization conditions may appear relatively stringent; however, the selected protocol was established based on a series of preliminary optimization experiments as well as previously published studies involving similar plant materials and tissue culture conditions.

**Comment 8:** *The use of GA3 in the germination medium and its potential residual effect on the explants was not addressed.*

**Response:**

 We appreciate the reviewer for raising this valuable point. We would like to clarify that **GA₃ (0.5 mg/L)** was used exclusively during the seed germination stage to promote dormancy breaking and enhance germination of sterilized seeds. Importantly, GA3 was not included in any subsequent tissue culture media used for callus induction or proliferation experiments.

***Third: Statistical Analysis and Interpretation***

**Comment 10:** *The ANOVA tables do not include F-values and p-values, and no multiple comparison letters are presented.*

**Response:**

 We sincerely thank the reviewer for this valuable observation, which has significantly contributed to improving the statistical clarity of the manuscript. In response, all relevant tables ( **Tables 2–5**) have been comprehensively revised to include the calculated **F-values** and corresponding **p-values** obtained from the analysis of variance ( **ANOVA**) for both the main effects and interaction effects.

 Additionally, to facilitate interpretation and enable clearer identification of statistically significant differences among treatment means, multiple comparison letters (a, b, c, etc.) have been incorporated based on the Least Significant Difference ( **LSD) test** at a significance level of **p ≤ 0.05**. These annotations have been added adjacent to the reported means to improve readability and statistical transparency.

**Comment 11:** *There is a major error in the Y-axis scale of Figure 1 (exceeding 100%), the error bars are not defined, and the high variability is not discussed.*

**Response:**

 Thank you for this important observation. Figure 1 has been revised to correct the Y-axis scaling so that the callus formation percentage does not exceed 100%. In addition, the type of error bars has now been clearly defined in the figure legend as standard error (SEM).

**Fourth: Supporting the Results and Conclusions**

**Comment 12:** *There is no justification for the term “synergistic efficiency” because the factors were tested in isolation and without a true interaction analysis.*

**Response:**

 We sincerely thank the reviewer for this insightful and scientifically important observation. We fully acknowledge that the use of the term **“**synergistic efficiency” was not sufficiently justified within the framework of the experimental design, as the investigated factors were evaluated in a sequential manner rather than through a full factorial interaction analysis.

 In response, we have carefully revised the terminology throughout the **title, main text, and conclusions** to ensure greater conceptual precision and alignment with the actual study design. Accordingly, the revised title now reads:

*“Evaluating the Efficiency of an Optimal Combination of Hormonal, Biological, and Physical Stimuli in Inducing and Promoting Callus Formation in* ***Lavandula angustifolia*** *Mill.”*

**Comment 13:** *The physiological concept of auxin action is incorrect.*

**Response:**

 We sincerely appreciate the reviewer for identifying this conceptual inaccuracy. In response, the relevant statements in both the **Introduction** and **Discussion** sections have been revised to reflect a more accurate physiological interpretation of auxin function in plant tissue culture.

**Comment 14:** *The justification for the superiority of B5 medium is superficial.*

**Response:**

 We sincerely thank the reviewer for this valuable observation, which enabled us to substantially strengthen the interpretation of our findings. In response, the **Discussion** section has been expanded to provide a more comprehensive physiological and nutritional justification for the superior performance of **B5 medium** relative to **MS medium** in supporting callus growth.

**Comment 15:** *The claim regarding secondary metabolite production is not supported by experimental analysis.*

**Response:**

 We sincerely thank the reviewer for this important observation and fully acknowledge that the original wording may have unintentionally overstated the scope of the present findings. Since no direct biochemical or metabolomic analyses were conducted to evaluate secondary metabolite production, we agree that such claims should not be presented as experimentally validated outcomes of this study.

**Comment 16:** *Callus quality assessment was limited to weight measurements only.*

**Response:**

 We sincerely appreciate the reviewer for this valuable suggestion, which has strengthened the evaluation of callus performance in the revised manuscript. In response, we have expanded the assessment of callus quality beyond biomass measurements by incorporating a **supplementary qualitative evaluation** of callus morphology under the most effective treatment conditions.

**Fifth: Figures and Image Quality**

**Comment 17:** *Figure 1 (Y-axis scale, error bars).*

**Response:**

 This issue has been fully addressed as described in **Comment 11**. Briefly, the **Y-axis scale** has been corrected to range from **0–100%**, and the **error bars** have been explicitly identified in the figure legend as representing the **standard error of the mean (±SE)**.

**Comment 18:** *Figure 2 is of low quality, has a blurred background, does not specify the treatment, and lacks a scale bar.*

**Response:**

 We sincerely thank the reviewer for this important observation. In response, **Figure 2** has been completely replaced with a **high-resolution image (300 DPI)** to improve visual clarity and presentation quality. The revised image was prepared using a **uniform background** to minimize visual interference and enhance specimen visibility

**Comment 19:** *Figure 3 does not specify whether the images correspond to MS or B5 medium.*

**Response:**

 We appreciate the reviewer for highlighting this lack of clarity. In response, the revised **Figure 3** has been updated to include **direct labels within each image panel**, positioned in the upper-right corner to clearly indicate the treatment condition (e.g., **“B5 + Chitosan 100 **mg/L”****).

**Comment 20:** *There is no photographic documentation of the 2,4-D + BA experiment.*

**Response:**

 We sincerely thank the reviewer for this valuable suggestion. In response, we have incorporated a **new **figure (Figure 4) into the revised manuscript, presenting representative photographic documentation of the optimized hormonal combination (1 mg/L 2,4-D + 2 mg/L BA **)**.

 This figure illustrates the morphological characteristics and quality of the resulting callus, including the **vigorous, sustainable, and morphologically uniform callus structure** that was subsequently selected for use in later experimental stages

 We sincerely hope that the revisions, clarifications, and additional analyses incorporated into the revised manuscript have adequately addressed all of the reviewer’s insightful comments and concerns. We are deeply grateful for the reviewer’s constructive feedback, which has significantly contributed to improving the scientific rigor, clarity, and overall quality of the manuscript.

 We trust that the revised version now meets the required scientific and publication standards, and we remain fully committed to implementing any further revisions or recommendations that may be suggested.

**Sincerely,**

**The Authors**

# Reviewer report: Reviewer response for version 1

**DOI:** 10.5256/f1000research.191471.r476656

**Published:** 2026-04-27

**Authors:** Duha Mysire Majeed

**Affiliations:** 1 Plant Biotechnology, Al-Nahrain University / Biotechnology Research center, Baghdad, Iraq

This study investigates the combined effects of hormonal, environmental, and biological factors on callus induction and growth in lavender. The topic is relevant and of interest in plant tissue culture; however, several aspects require improvement to enhance the scientific quality of the manuscript.

**Major comments:**

1. The manuscript requires substantial language editing to improve clarity and scientific writing style. Several sentences are repetitive and lack precision.
2. The novelty of the study is limited, as it mainly focuses on optimization using well-established factors such as 2,4-D, BA, nutrient media, and chitosan. The authors should better highlight the originality of their contribution.
3. The experimental design is not sufficiently detailed. The authors should clearly describe the explant type, treatment structure, and factorial design to ensure reproducibility.
4. The statistical analysis needs to be strengthened. The manuscript lacks detailed ANOVA results, p-values, and a clear explanation of statistical significance.
5. The discussion section should be improved by providing deeper interpretation of the results and better comparison with recent literature.

**Minor comments:**

- Ensure consistency in units and formatting (e.g., mg L⁻¹).
- Improve figure quality and labeling.
- Correct grammatical and typographical errors.

**Conclusion:**

 The conclusions are generally supported by the results but are somewhat overstated and should be moderated.

**Recommendation: Major Revision**

Is the work clearly and accurately presented and does it cite the current literature?

Partly

If applicable, is the statistical analysis and its interpretation appropriate?

Yes

Are all the source data underlying the results available to ensure full reproducibility?

Yes

Is the study design appropriate and is the work technically sound?

Partly

Are the conclusions drawn adequately supported by the results?

Partly

Are sufficient details of methods and analysis provided to allow replication by others?

Partly

Reviewer Expertise:

Plant Biotechnology, Plant Tissue Culture, Molecular Biology, Plant Genetic Engineering

I confirm that I have read this submission and believe that I have an appropriate level of expertise to confirm that it is of an acceptable scientific standard, however I have significant reservations, as outlined above.

# Response

**Published:** 2026-04-30

**Authors:** MOHAMMED ABDULGAFOR

**Affiliations:** biotechnology, University of Anbar, Ramadi, Al Anbar Governorate, Iraq

Response to Reviewer Comments

 We sincerely thank the reviewer for their careful evaluation of our manuscript and for providing constructive and insightful comments that have helped us improve the quality of the work.

**Comment 1: **Language editing

- We appreciate this important remark. The manuscript has undergone professional language editing prior to submission; however, we acknowledge that some sections may still require further refinement. Accordingly, we have carefully revised the text to improve clarity, reduce repetition, and enhance the overall scientific style.

**Comment 2:** Limited scientific novelty

- We thank the reviewer for this valuable observation. While the study employs established factors such as 2,4-D, BA, nutrient media, and chitosan, its novelty lies in the integrated evaluation of multiple interacting variables within a single experimental framework. Specifically, the originality of this study is reflected in: The combined assessment of hormonal, environmental (light/dark), and biological (chitosan) factors and their interactive effects on callus induction and growth in lavender. Demonstrating that plant responses to identical growth regulator concentrations vary depending on tissue type and treatment combinations, which may explain inconsistencies reported in previous studies. We have revised the Introduction and Discussion sections to more clearly highlight these contributions.

**Comment 3:** Experimental design details

- We appreciate the reviewer’s concern regarding reproducibility. The manuscript has been revised to provide clearer and more detailed descriptions of the experimental design. Specifically: The explant type (leaf tissue) is now explicitly stated. The treatment structure has been clarified, including: Four concentrations of 2,4-D (0, 1, 2, 3 mg L⁻¹) under two light conditions. A factorial combination of 2,4-D (1, 2, 3 mg L⁻¹) and BA (0, 1, 2 mg L⁻¹). Additional clarification has been added regarding replication and experimental setup. We believe these revisions sufficiently improve reproducibility.

**Comment 4:** Statistical analysis

- We thank the reviewer for highlighting this point. The statistical methodology has been clarified in the revised manuscript, including: The use of a Completely Randomised Design (CRD) with ten replicates. A factorial arrangement of treatments. Mean separation using the LSD test at P ≤ 0.05. P-values have been included within the results where appropriate. To maintain manuscript conciseness, full ANOVA tables were not included in the main text; however, we are willing to provide them as supplementary material upon request.

**Comment 5:** Discussion of the results

- We appreciate this constructive suggestion. The Discussion section has been revised to provide a deeper interpretation of the findings and stronger linkage to existing literature. Due to the limited number of studies specifically addressing lavender callus induction under similar combined conditions, we have incorporated relevant and recent studies from related species within the same family (Lamiaceae), while clearly acknowledging this limitation. This also underscores the importance of our study in addressing a gap in the literature. Additional minor comments All minor issues raised by the reviewer have been addressed: Units and formatting (e.g., mg L⁻¹) have been standardised. Figures have been improved in clarity and labeling. Grammatical and typographical errors have been corrected. Conclusion We have revised the conclusion to ensure it accurately reflects the results without overstatement, in line with the reviewer’s recommendation.
